# Physiological Mechanisms Driving Microcirculatory Enhancement: the Impact of Physical Activity

**DOI:** 10.31083/RCM25302

**Published:** 2025-02-19

**Authors:** Jianyu Li, Guochun Liu, Dong Zhang, Keying Zhang, Chunmei Cao

**Affiliations:** ^1^Division of Sports Science and Physical Education Tsinghua University, Tsinghua University, 100084 Beijing, China; ^2^College of Exercise Medicine, Chongqing Medical University, 400331 Chongqing, China; ^3^Institute of Artificial Intelligence in Sports, Capital University of Physical Education and Sports, 100091 Beijing, China; ^4^Department of Physical Education, Southeast University, 210012 Nanjing, Jiangsu, China

**Keywords:** microcirculation, physical activity, rehabilitation, physiological mechanisms

## Abstract

**Background:**

Physical activity induces many beneficial adaptive changes to blood vessel microcirculation, ultimately improving both health and exercise performance. This positions it an effective non-pharmacological therapeutic approach for the rehabilitation of patients with various chronic diseases. Understanding the impact of different types of physical activities on microcirculation and elucidating their physiological mechanisms is crucial for optimizing clinical practice.

**Methods:**

A comprehensive literature search was performed across multiple databases including PubMed, EBSCO, ProQuest, and Web of Science. Following a rigorous screening process, 48 studies were selected for inclusion into the study.

**Results:**

Existing studies demonstrate that various forms of physical activity facilitate multiple positive adaptive changes at the microcirculation level. These include enhanced microvascular dilation—driven by endothelial cell factors and mechanical stress on blood vessels—as well as increased capillary density. The physiological mechanisms behind these improvements involve the neurohumoral regulation of endothelial cell factors and hormones, which are crucial for these positive effects. Physical activity also ameliorates inflammation markers and oxidative stress levels, upregulates the expression of *silent information regulator 2 homolog 3*, genes for hypoxia-inducible factors under hypoxic conditions, and induces favorable changes in multiple hemodynamic and hemorheological parameters. These structural and functional adaptations optimize myocardial blood flow regulation during exercise and improve both oxygen transport and utilization capacity, which are beneficial for the rehabilitation of chronic disease patients.

**Conclusions:**

Our provides a reference for using physical activity as a non-pharmacological intervention for patients with chronic conditions. This framework includes recommendations on exercise types, intensity, frequency, and duration. Additionally, we summarize the physiological mechanisms through which physical activity improves microcirculation, which can inform clinical decision-making.

## 1. Introduction

Microcirculation refers to physiological processes within small blood vessels, such as arterioles, venules, and capillaries, which are essential for energy metabolism, substance exchange, and waste removal [1]. Microcirculatory dysfunction plays a crucial role in the pathophysiology of aging and many chronic diseases including diabetes, hypertension and heart failure [2]. This dysfunction is characterized by reduced microvascular blood perfusion, increased oxygen deficit accumulation, and impaired microvascular endothelial dilation [3, 4, 5]. Given the critical role of microcirculation in oxygen transport and energy metabolism, improving microcirculatory function has emerged as a significant area of interest for researchers.

Physical activity is an effective non-pharmacological therapy for improving microcirculatory function [6, 7, 8]. Numerous studies have demonstrated that aerobic exercise, resistance training, hypoxic interventions, and functional physical activity are effective strategies for enhancing microcirculatory function and reducing the risk of microvascular dysfunction associated with aging and chronic diseases [9, 10, 11, 12]. Notably, acute high-intensity exercise in sedentary individuals can lead to microvascular endothelial dysfunction, whereas resistance-trained individuals maintain vasodilation under similar conditions, highlighting the plasticity of microvascular function in response to resistance training. Additionally, hypoxic interventions, blood flow restriction, vibration training and other functional physical activities have shown positive effects on microcirculatory function in clinical interventions [13, 14].

The physiological mechanisms by which physical activity improves microcirculatory function encompass a variety of biological pathways that are still being explored. Research suggests that one mechanism involves neurohumoral regulation, where physical activity enhances microcirculatory function by modulating the secretion of endocrine and endothelial factors [15, 16, 17]. Additionally, research highlights the reduction of inflammatory factors and oxidative stress levels as another pathway for ameliorating microcirculatory dysfunction [18]. Additional evidence indicates that physical activity promotes increased vascular shear stress and mitochondrial biogenesis, which along with improvements in mitochondrial dynamics mediated by adenosine 5^′^-monophosphate-activated protein kinase (AMPK), enhance microvascular function by improving hemodynamics [19]. Furthermore, cellular hypoxia stimulation and erythrocyte membrane deformability may be potential physiological mechanisms underlying the improvement in microcirculatory function [20].

A recent study has demonstrated the capacity of physical activity to regulate vascular function and ameliorate microcirculatory dysfunction through various physiological pathways. Nevertheless, the scope and implications of these findings are subject to ongoing debate within the scientific community [21]. This review aims to delineate the most recent advancements, providing a comprehensive overview of the effects and physiological mechanisms by which physical activity influences microcirculatory function. This article explores the impact of different forms of exercise, including aerobic exercise, resistance training, hypoxic interventions, and functional training on microcirculation. Further we discuss the biological mechanisms contributing to improvements in microcirculatory function and highlight the importance of these mechanisms and their role as non-pharmacological interventions that promote health and aid in disease recovery.

## 2. Materials and Methods

**Fig. 1.**
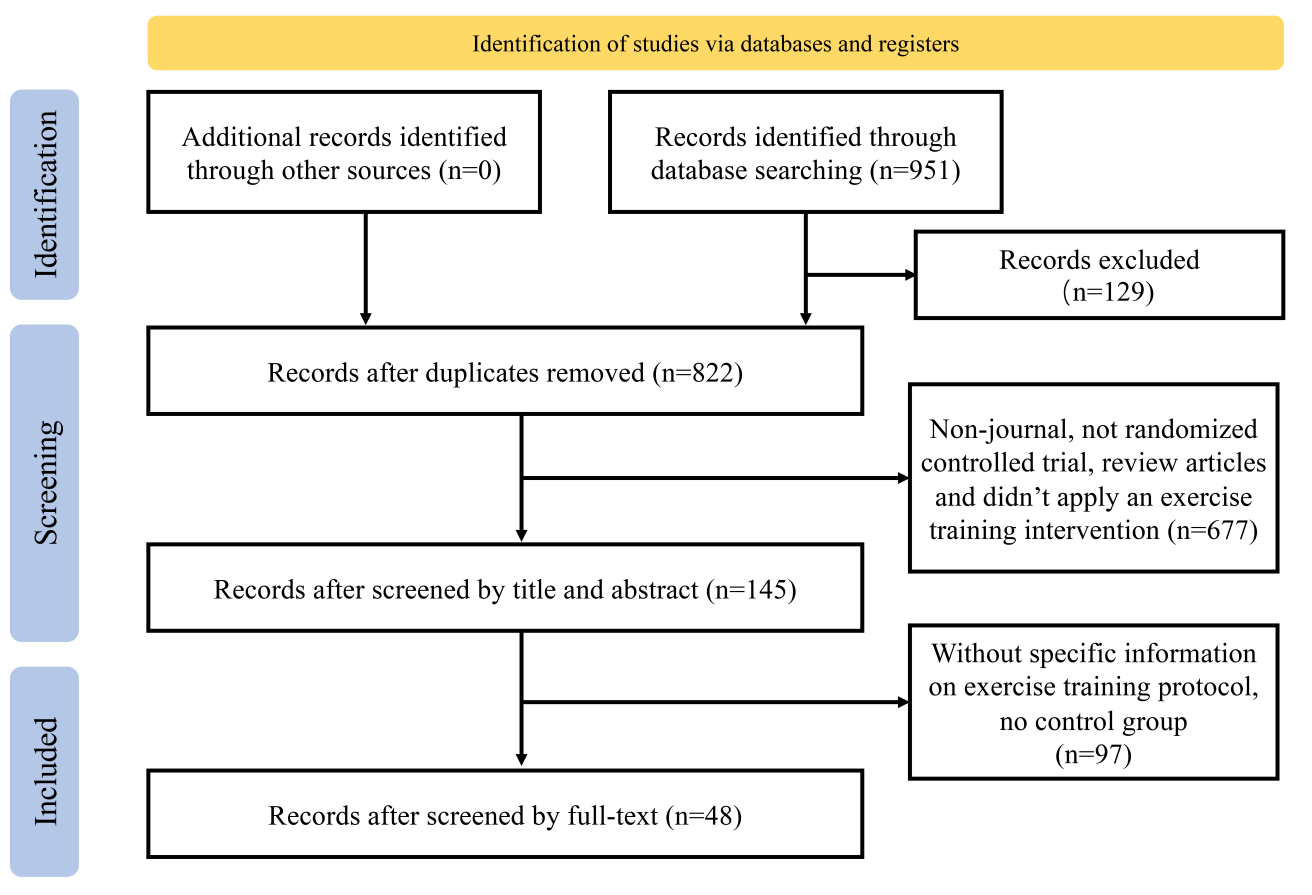
**PRISMA search strategy and article selection for microcirculation studies**. PRISMA, the Preferred Reporting Items for Systematic Reviews and Meta-Analyses.

This review was conducted by querying several databases—PubMed, EBSCO, ProQuest, and Web of Science databases using the search terms “exercise”, “physical activity”, “physical exercise”, “acute exercise”, “isometric exercise”, “aerobic exercise”, “exercise training”, “microvascular blood flow” and “microvascular circulation” for English-language articles published between January 1, 2014, and May 31, 2024. This review follows the Preferred Reporting Items for Systematic Reviews and Meta-Analyses (PRISMA) guidelines (Fig. 1). Two researchers independently performed the literature search and screening. In cases of disagreement, further discussion was conducted to reach a consensus. Data extraction from the selected studies was also performed independently by both researchers.

**Fig. 2.**
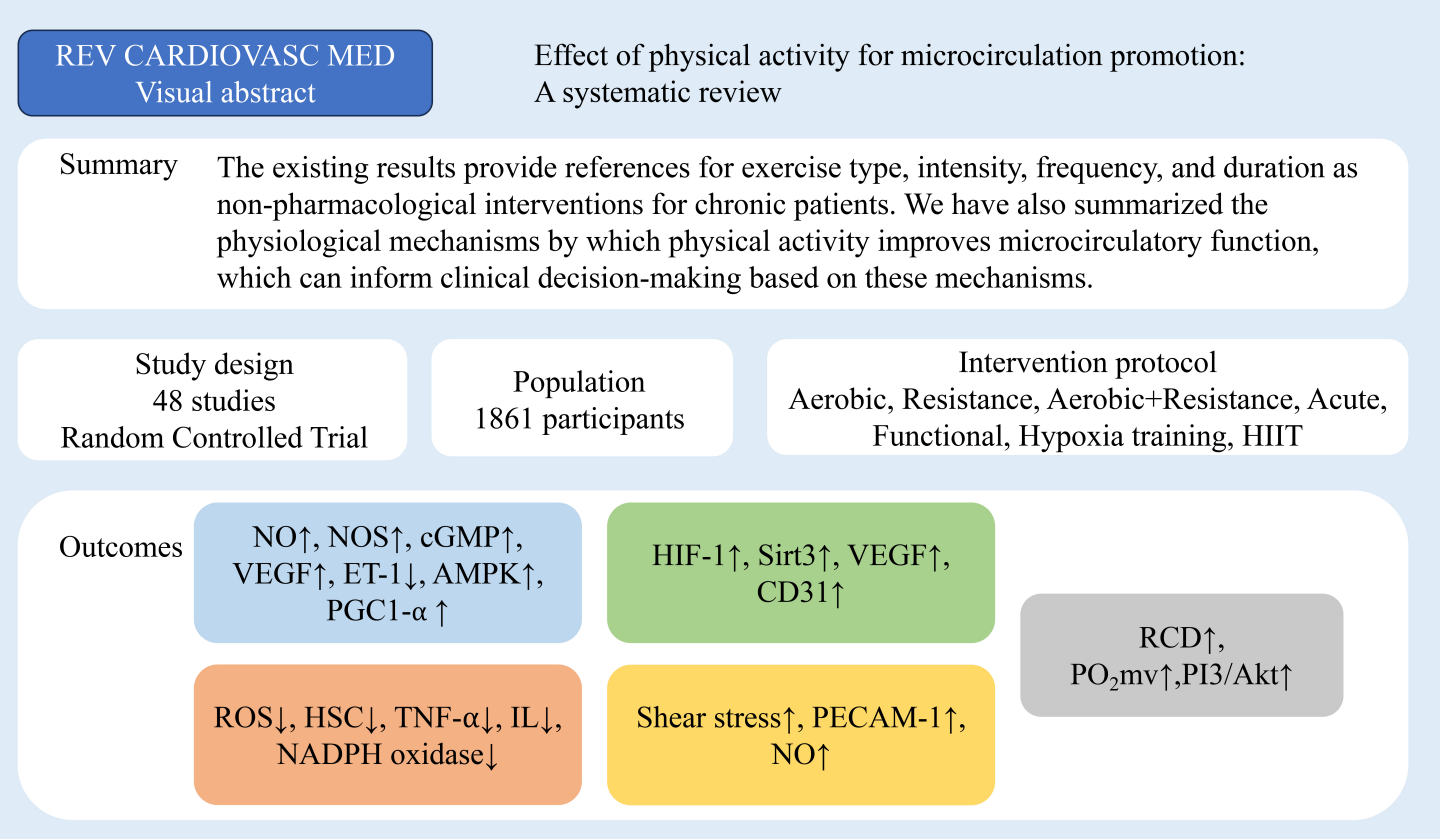
**Study selection for evaluating the impact of physical activity on microcirculatory function**. REV CARDIOVASC MED, Reviews in cardiovascular medicine; HIIT, high intensity interval training; NO, nitric oxide; NOS, nitric oxide synthase; cGMP, cyclic guanosine monophosphate; VEGF, vascular endothelial growth factor; ET-1, endothelin-1; AMPK, adenosine5^′^-monophosphate-activated protein kinase; PGC1-α, peroxisome proliferator-activated receptor gamma coactivator-1alpha; ROS, reactive oxygen species; HSC, hepatic stellate cells; TNF-α, tumor necrosis factor-α; IL, interleukin; NADPH, nicotinamide adenine dinucleotide phosphate; HIF-1, hypoxia inducible factor-1; Sirt3, silent information regulator 2 homolog 3; CD31/PECAM-1, cluster of differentiation 31/platelet endothelial cell adhesion molecule-1; RCD, red cell deformability; PO_2_mv, muscle microvascular oxygenation; PI3K/Akt, phosphoinositide 3-kinase/Protein kinase B; ↑, positive; ↓, negative.

The inclusion criteria for the literature were as follows: (1) studies that utilized a randomized controlled trial (RCT) design to evaluate changes in microcirculatory function before and after physical activity interventions; (2) studies published in English between January 2014 and May 2024. The exclusion criteria were: (1) studies lacking a control group; (2) studies without specific information on microcirculatory function assessment or details of the physical activity intervention; (3) prospective studies. The detailed flowchart of the study selection process is presented in Fig. 2.

## 3. Results and Discussion

Microcirculatory dysfunction is influenced by the interaction of several signaling pathways, and various types of physical activity demonstrate therapeutic potential as non-pharmacological interventions through multi-target effects [22, 23]. These interventions are notable for their multi-target effects, capable of alleviating microcirculatory dysfunction and restoring endothelial homeostasis. Specifically, physical activities promote microvascular dilation and exert anti-inflammatory and antioxidant effects, which collectively improve hemodynamics and hemorheology. However, the clinical efficacy of these activities, including aerobic exercise, resistance exercise, hypoxia training, functional exercise, high intensity interval training (HIIT), and moderate intensity continuous (MICT), shows variability (Table 1, Ref. [8, 9, 10, 11, 12, 13, 14, 15, 16, 17, 18, 19, 20, 24, 25, 26, 27, 28, 29, 30, 31, 32, 33, 34, 35, 36, 37, 38, 39, 40, 41, 42, 43, 44, 45, 46, 47, 48, 49, 50, 51, 52, 53, 54, 55, 56, 57, 58]). This review categorizes the physiological mechanisms by which physical activity influences microcirculatory function into six key areas: neurohumoral regulation, anti-inflammation, antioxidation, hemodynamics, adaptation to hypoxic environments, and hemorheology enhancements.

**Table 1.** **Summary of studies on the effects of various types of physical activity on microcirculation**.

Type	Study	Number	Duration	Protocol	Result		
					Positive		Negative
Healthy animal experimentation							
Aerobic	Herrera *et al*., 2016 [24]	76	8 weeks	Per day/1 h 60% of T_max_ run	E: Capillary density; Capillary-to-fibers ratio; VEGF		
	Hirai *et al*., 2014 [25]	22 (E: n = 11; C: n = 11)	6 to 7 weeks	5 times a week/1 h treadmill run	E: VO_2peak_; Citrate synthase activity; Speed of PO2mv fall during contractions markedly slowed		
	Leardini-Tristão *et al*., 2017 [26]	20 (E: n = 10; C: n = 10)	7 days	Per day/30 min 60% VO_2max_ treadmill run	E: Functional capillary density	E: Microvascular cerebral blood flow	E: Leukocyte adhesion; *NADPH* oxidase gene expression
	Mazur-Bialy *et al*., 2017 [15]	Not state (E: n = 8–15; C: n = 8–15)	6 weeks	Per day/Wheel running	E: Colonic blood flow; Plasma irisin; WAT concentrations of adiponectin		E: TNF-α; IL-6; MCP-1; IL-1β; Liptin levels;
					C: Colonic tissue weight; TNF-α; IL-6; MCP-1; IL-13		C: Colonic blood flow
	Robinson *et al*., 2017 [27]	Not state	2 weeks	Per day/6 km run	E: SOD isoform expression	E: FID; Superoxide levels	E: NOX II protein expression; Sensitivity to Ang II
					C: Superoxide levels		C: FID
	Leardini-Tristão *et al*., 2020 [28]	53 (E: n = 12; C: n = 11; CCHE: n = 15; CCHC: n = 15)	12 weeks	3 times a week/ 30-min 60% V_max_ treadmill running	E: Functional capillary density; Synaptic proteins expression in the brain; Astrocytes vessel coverage; Structural capillaries		E: Leukocyte rolling; Microglial activation
	Yuan *et al*., 2021 [29]	60 (E+TAC: n = 20; TAC: n = 20; C: n = 20)	10 weeks	5 times a week/60 min 15 m/min treadmill run	E: Number of skeletal muscle capillaries; mRNA and Protein levels of VEGF; Skeletal muscle mass		E: Blood pressure
	Zhang *et al*., 2022 [19]	25 (E+ALPR: n = 5; ALPR: n = 5; E: n = 5; MI: n = 5; C: n = 5)	6 weeks	5 times a week/Treadmill run	E: VEGF; FGF; Microangiogenesis; ATP		E: TSP-1; Inhibited MAPK signaling pathway; ADP; AMP
	Shin *et al*., 2023 [30]	18 (E: n = 9; C: n = 9)	5 months	Voluntary wireless running wheel run	E: Capilary flux; Capilary RBC speed; Microvascular oxygenation; Cortical microvascular density	E: RBC line-density; Cortical hemodynamic response to functional activation	E: Coeffcient of variation of capillary RBC flux
HIIT	Marques Neto *et al*., 2020 [31]	23 (HIIT: n = 6; HFD: n = 6; HIIT+HFD: n = 5; C: n = 6)	4 weeks	5 times a week/Treadmill running consisted of 7 times 3-min at 85% of VO_2max_ and 3-min intervals at 60% of VO_2max_	E: VO_2max_; Contractility and relaxation index; Capillary diameter; Capillary functional density; Mitochondrail swelling		
Hypoxia training	Ma *et al*., 2022 [13]	24 (E: 6/3-TYP: 6/E+3-TYP: 6/C: 6)	6 weeks	6 times a week/Lived hypoxic tent for 8 h with an oxygen concentration of 13.8% and treadmill run in normoxia	E: MBP; H-MBP; Sirt3; Ang II; NO; CD31; VEGF; Mitochondrial volume		
Chronic disease animal model							
Aerobic	Ranjbar *et al*., 2017 [20]	30 (E: n = 10; C: n = 10; Sham: 10)	10 weeks	5 times a week/50 min 60% VO_2max_ treadmill run	E: Slow twitch muscle capillary density; Capillary to fiber ratio at slow twitch muscle; Fast twitch muscle arteriolar density; HIF-1	E: Slow twitch muscle (VEGF; FGF-2; Angiostatin gene expression); Fast twitch muscle (FGF-2; TGF-β)	E: Slow twitch muscle (TGF-β); Fast twitch muscle (VEGF; Angiostatin)
	Lapi *et al*., 2023 [9]	33 (E: n = 11; D: n = 11; E+D: n = 11)	6 weeks	3 times a week/Moderate exercise	E: Microvascular permeability; Perfused capillary length		E: ROS; Number of leukocytes adhering to the venular walls
	Sytha *et al*., 2023 [32]	50 (E: n = 25; C: n = 25)	14 weeks	5 times a week/Treadmill run	E: Citrate synthase activity; Endothelium-dependent dilation	E: Smooth muscle function; Bradykinin-mediated dilation; Basal superoxide levels	E: Bradykinin sensitivity
	Rodrigues *et al*., 2022 [33]	20 (E: n = 10; C: n = 10)	12 weeks	3 times a week/75%–80% VO_2max_ treadmill run	E: Hepatic lipid peroxidation; antioxidant enzyme catalase activity; Nitrite level; CAT		E: Hepatic steatosis and fibrosis; Leukocyte rolling and adhesion in the liver and adipose tissue microcirculation; AGE deposition; RAGE protein expression
Resistance	Guimarães-Ervilha *et al*., 2023 [10]	32 (E: n = 8; C: n = 8; PAHE: n = 8; PAHC: n = 8)	30 days	Per day/Climbed 1.1 m high ladder with an inclination of 80° three times with resting intervals of 2 min	E: NO	E: SOD; CAT; FRAP; CP	E: GST; MDA
Healthy people							
Aerobic	Alkhatib *et al*., 2014 [8]	15	8 weeks	2 times a week/20 min Moderate cycle	E: VT, CVC	E: Time to reach maximum perfusion	-
HIIT	Solianik *et al*., 2021 [34]	11	1 time	3 × 90% HR_max_ three-time rounds	E: Superior and inferior temporal venules dilatation	E: MAP	E: Arteriolar-to-venular diameter ratio;
Resistance	Durand *et al*., 2015 [35]	54 (Exercise trained: n = 33; C: n = 21)	1 time	≥4 sets of 10 repetitions leg bench press with free weights	ET: Flow-mediatied dilation	ET: ACh-mediated vasodilation matained	C: Flow-mediated dilation; ACh-mediated vasodilation
	Tryfonos *et al*., 2023 [36]	11 (E: n = 6; C: n = 5)	1 time	30 min continuous rhythmic handgrip exercise	E: Radial artery mean; Antegrade shear rate; Mean arterial pressure; eNOS Ser1177	E: PECAM-1; PECAM-1 Tyr713	C: Mean arterial pressure
						C: Radial artery mean; Antegrade shear rate; eNOS Ser1177; PECAM-1; PECAM-1 Tyr713	
Acute	Stupin *et al*., 2018 [37]	38 (Exercise trained: n = 20; C: n = 21)	1 time	5 × 4 min submaximal grades and a single maximal grade	C: ACh-induced dilation; FRAP	E: SNP-induced dilation; TBARS; FRAP	E: PORH; ACh-induced dilation
						C: PORH; SNP-induced dilation; TBARS	
Functional	Jeffries *et al*., 2018 [11]	20 (E: n = 10; C: n = 10)	7 days	4 × 5 min lower limb ischemic preconditioning	E: Muscle oxidative capacity	C: Resting muscle oxygen consumption; Deoxygenated hemoglobin; Muscle oxidative capacity	E: Resting muscle oxygen consumption; Deoxygenated hemoglobin
Hypoxia training	Meng *et al*., 2019 [12]	20 (E: n = 12; C: n = 8)	4 weeks	6 times a week/3000 m altitude hypoxia training	E: VO_2peak_; MPO; P_4_; CMBC; PORH; EPO; HIF; NO; ET	E: eNOS; PGI2; VEGF	
						C: VO_2peak_; MPO; P_4_; CMBC; PORH; EPO; HIF; NO; eNOS; ET; PGI2; VEGF	
Clinical studies on populations with chronic conditions							
Aerobic	Boa *et al*., 2014 [38]	108 (E: n = 44; C: n = 64)	4 weeks	5 times a week/1 h light, moderate run	E: Area of the rough endoplasmic		C: endothelium-dependent vasodilatation
					Reticulum; endothelium-dependent vasodilatation		
	Moraes *et al*., 2016 [18]	22	12 weeks	4 times a week/30–60 min 40% HRR walk or run	E: Capillary density	E: Skin microvascular vasodilation responses induced by either endothelial-dependent or endothelial-independent vasoactive drugs	E: Uric acid; IL-6
	Borges *et al*., 2018 [16]	34 (High frequency E: n = 23; Low frequency E: n = 11)	6 weeks	HF: ≥2 times a week; LF: ≤2 times a week; Cardiac rehabilitation	HF: Skin microvascular blood flow; Cutaneous vascular conductance (Higher than LF); NO bioavailability	HF: Catalase activity	HF: Lipid peroxidation
					LF: Skin microvascular blood flow; Cutaneous vascular conductance	LF: Catalase activity	LF: Lipid peroxidation; NO bioavailability
	Szyguła *et al*., 2020 [39]	48 (E: n = 24; C: n = 24)	8 weeks	3 times a week/30–40 min Aerobic march training	E: Regular flow; PRH_max_; TH_max_; Signal strength of endothelic rhythm		E: Time to achieve PRH_max_; Time to achieve TH_max_; Heart rhythm; Signal strength of the neurogenic rhythm
Aerobic+ resistance	Marini *et al*., 2019 [40]	30 (E: n = 15; C: n = 15)	12 weeks	2 times a week/ 60-min 50%–70% reserve heart rate treadmill running or cycling and 30-min 50% 1RM muscular strength exercise.	E: HDL-cholesterol; PORH; VO_2max_; AH		E: Fasting blood glucose; Serum HbA_1c_
	Vinet *et al*., 2018 [41]	62 (HR: n = 17; HE: n = 21; ME: n = 24)	6 months	HR: High resistance+moderate endurance; HE: High endurance+moderate resistance; ME: moderate endurance+ moderate resistance	FMD (in % and relative to peak shear rate)	Endothelium-dependent and endothelium-independent function of the forearm skin microcirculation	IL-6
HIIT	Hollekim-Strand *et al*., 2014 [42]	37 (E: n = 20; C: n = 17)	12 weeks	3 times a week/40 min for 4 intervals (3 min 90%–95% VO_2max_)	E: Diastolic function; Systolic function; Global strain rate; FMD		E: C-reactive protein level; HbA_1c_
	Suryanegara *et al*., 2019 [43]	26 (E: n = 13; C: n = 13)	12 weeks	3 times a week/5 × 3 min 16–17 (Borg Rating: very hard) cycle	E: Peak exercise arterial-venous oxygen difference	E: Glycated hemoglobin;	E: Cardiac output
	Streese *et al*., 2020 [44]	74 (E: n = 40; C: n = 34)	12 weeks	3 times a week/45 min 80%–90% Nordic Walking-based HIIT	E: Retinal arteriolar diameter; Arteriolar-to-venular diameter ratio		E: Venular diameter; Mitochondrial adaptor *p66*^𝑆ℎ𝑐^ gene expression
	Streese *et al*., 2020 [45]	69 (E: n = 33; C: n = 36)	12 weeks	3 times a week/4 × 4 min at 80–90% HR_max_ with 3 min of active recovery	E: ADmax; AFarea; VO_2peak_	E: VDmax; VFarea	
	Mitropoulos *et al*., 2018 [46]	31 (Cycling E: 10/Arm cranking E: 10/C: 11)	12 weeks	2 times a week/30-min consist of 30 s 100% exercise followed by 30 s rest, alternating.	E:* ∆*TcpO2; Endothelial-dependent vasodilation	E: Cutaneous vascular conductance;	E; Raynaud’s phenomenon
HIIT/MICT	Mitranun *et al*., 2014 [17]	43 (E_1_: n = 14; E_2_: n = 14; C: n = 15)	12 weeks	3 times a week/HIIT: 30–40 min for 4–6 intervals (85% VO_2max_ 1 min following 50% VO_2max_ 4 min active rest); MICT: 30–40 min for 4–6 intervals (50%–65% VO_2max_)	E: NO; Glutatione peroxidase; FMD; Ratio of maximal to resing cutaneous blood flow	E: Resting and maximal crtaneous blood flow	E: HbA_1c_; Fasting glucose concentration; Insulin resetance
	Hwang *et al*., 2019 [47]	58 (E_1_: n = 23; E_2_: n = 19; C: n = 16)	8 weeks	4 times a week/HIIT: 4 × 4-min intervals 90% HR_peak_; MICT: 32 min at 70% HR_peak_	E: VO_2peak_		
	Mortensen *et al*., 2019 [48]	21 (E_1_: n = 11; E_2_: n = 10)	11 weeks	3 times a week/HIIT: 20-min consist of 10 times 1 min at 95% W_peak_ and 1 min of active recovery; MICT: 40-min	E_1_: eNOS	E: Skeletal muscle capillary area; Capillary density	E_2_: Thickness of the basement membrane
					E_2_: Capillary-to-fiber ratio; Mean arithmetic thickness of endothelial cells; Capillary lumen	E_1_: Capillary-to-fiber ratio; Mean arithmetic thickness of endothelial cells; Capillary lumen; Thickness of the basement membrane; VEGF; SOD-2; NADPH oxidase	
						E_2_: eNOS; VEGF; SOD-2; NADPH oxidase	
	Baasch-Skytte *et al*., 2020 [49]	44 (E_1_: n = 23; E_2_: n = 21)	10 weeks	3 times a week/HIIT: 5 consecutive 1 min exercise periods divided into 30, 20 and 10 s at low, moderate and maximal intensity; MICT: 50 min moderate intensity continuous cycle (60%–75% HR reserve)	E: VO_2max_; Plasma norepinephrine	E: Fasting plasma glucose; C-petide	E: HbA_1c_;
						E_1_: AUC_glucose_	E_2_: AUC_glucose_;
	Gildea *et al*., 2021 [50]	28 (E_1_: n = 9; E_2_: n = 10; C: n = 9)	12 weeks	3 times a week/HIIT: 10 × 1 min 90% HR_max_ cycle/MICT: 50 min (80%∼90%VT) cycle	E: VO_2peak_; Muscle fractional O_2_ extraction	E: τVO_2⁢p_; End-exercise VO_2_ amplitude; Functional VO_2_ gain	E: HbA_1c_
	Li *et al*., 2022 [51]	37 (E_1_: n = 13; E_2_: n = 12; C: n = 12)	12 weeks	5 times a week/HIIT: 8 × 1 min 80%–95% cycle; MICT: 30 min 50%–70% cycle	E: VO_2max_		E: HbA_1c_
HIIT/END	Winding *et al*., 2018 [52]	32 (E_1_: n = 13; E_2_: n = 12; C: n = 7)	11 weeks	3 times a week/20 min for 10 intervals (95% of peak workload cycling following 1 min active rest)	E: VO_2peak_	E: Oral glucose tolerance test	E: HbA_1c_; Glycaemic variability; Homeostasis model assessment of insulin resistance
Acute	Tzanis *et al*., 2016 [53]	16 (Chronic heart failure: n = 8; C: n = 8)	-	Maximal exercise	CHF: Oxygen consumption rate	C: StO_2_	CHF: StO_2_
					C: Oxygen consumption rate		
	Zheng *et al*., 2021 [54]	48 (DM: n = 16; DM+ulcer: n = 16; C: n = 16)	5 min	Isometric ankle plantarflexion exercise			SBMF: C>DM>DM+uclear
Functional	Valensi *et al*., 2022 [14]	16	20 min	Diastole synchronized compressions/decompressions	E; CBF; AUC5_min_		E: Plasma glucose
		38	12 weeks	3 times a week/60 min diastole-synchronized compressions/decompressions	E: AUC5_min_; HDL	E: FMD; Brachial artery diameter; VT1	E: LDL-cholesterol; Non-HDL cholesterol; Triglycerides
	Baker *et al*., 2017 [55]	64 (E: n = 29; C: n = 35)	3 months	3 times a week/treadmill walk with grade	E: Calf muscle blood flow; Oxygen extraction	E: Recovery half-time for hemoglobin/myoglobin desaturation	
Mixed-mode intense exercise	Gaffney *et al*., 2021 [56]	24 (E: n = 12; C: n = 12)	10 weeks	3 times a week/20 min intensive interval cycling; 2 times a week/20 min resistance training	E: Basal and insulin-stimulated microvascular perfusion; Skeletal muscle mitochondrial; Capillary density		E; PCG-1α; Citrate synthetase
Resistance	Yang *et al*., 2023 [57]	32 (E: n = 16; C: n = 16)	12 weeks	2 times a week/Five upper exercises in a circuit row for three circles	E; MBP; CMBC; AVBC; SOD; GSH-PX		E: MDA; CAT
	Mitropoulos *et al*., 2019 [58]	32 (E: n = 16; C: n = 16)	12 weeks	2 times a week/Five upper exercises in a circuit row for three circles	E: VO_2peak_; *∆*TcpO2; *∆*TcpO2_max_; Time to peak endothelial-dependent reactivity; ACh Tmax; Endothelial-independent function	E: endothelial-dependent function	

Note: E, exercise group; n, sample size; C, control group; VO_2peak_, oxygen uptake peak; PO_2_mv, muscle microvascular oxygenation; VO_2max_, maximum oxygen uptake; TNF-α, tumor necrosis factor-alpha; IL-6, interleukin-6; MCP-1, monocyte chemoattractant protein-1; IL-1β, interleukin-1β; SOD, superoxide dismutase; NADPH, nicotinamide adenine dinucleotide phosphate; NOX, NADPH oxidase; CCHE, coronary heart disease exercise group; CCHC, coronary heart disease control group; HIIT, high intensity interval training; TAC, transverse aortic constriction; ALPR, alprostadil; MI, myocardial infarction; HFD, high-fat diet; TYP, triazolyl pyridine; TSP-1, thrombospondin-1; RBC, red blood cell; Sirt3, silent information regulator 2 homolog 3; Ang II, angiotensin II; NO, nitric oxide; CD31, cluster of differentiation 31; HIF-1, hypoxia-inducible factor 1; TGF-β, transforming growth factor-β; ROS, reactive oxygen species; AGE, advanced glycation end-products; PAHE, pulmonary arterial hypertension exercise group; PAHC, pulmonary arterial hypertension control group; HR, heart rate; VT, ventilatory threshold; CVC, cardiac vascular conductance; RAGE, receptor for advanced glycation end-products; ET, endothelin; eNOS, endothelial nitric oxide synthase; SNP, single nucleotide polymorphism; HRR, heart rate reserve; Ser1177, anti-phospho-eNOS ser1177; HIF, hypoxia inducible factor; PGI2, prostacyclin I2; HE, high intensity exercise group; ME, moderate intensity exercise group; HF, high frequency group; LF, low frequency group; HDL, high-density lipoprotein; HbA_1c_, hemoglobin A1c; MICT, moderate intensity continuous training; DM, diabetes mellitus; CHF, chronic heart failure; LDL, low-density lipoprotein; PCG-1α, peroxisome proliferator-activated receptor gamma coactivator 1-alpha; ACh, acetylcholine; PECAM, platelet endothelial cell adhesion molecule; FRAP, ferric reducing ability of plasma; TBARS, thiobarbituric acid reactive substances; MPO, max power output; P_4_, power at blood lactic acid of 4 mmol/L; CMBC, concentration of moving blood cells; 1RM, one-repetition maximum; PORH, post-occlusive reactive hyperemia; EPO, erythropoietin; *p66*^𝑆ℎ𝑐^, p66^Shc^ adaptor protein; ΔTcpO2, transcutaneous oxygen tension; MAP, mean arterial pressure; WAT, white adipose tissue; FID, flow-induced dilation; FGF, fibroblast growth factor; MAPK, mitogen-activated protein kinase; ATP, adenosine triphosphate; ADP, adenosine diphosphate; AMP, adenosine monophosphate; MBP, microcirculatory blood perfusion; H-MBP, blood perfusion response upon heating stimulation; PRH_max_, post-occlusive reactive hyperemia maximum; TH_max_, thermal hyperemia maximum; AUC_glucose_, plasma glucose area under the curve; CAT, catalase; CP, carbonylated proteins; GST, glutathione S-transgerase; MDA, malondialdehyde; StO_2_, oxygen saturation; ADmax, maximal arteriolar; AFarea, area under the arteriolar; VDmax, maximal venular; VFarea, area under the venular; AH, area of hyperaemia; CBF, cutaneous blood flow; AUC5_min_, CBF area under the curve after ACh administration; SMBF, skeletal muscle blood flow; FMD, flow-mediated dilatation; τVO_2⁢p_, pretraining time constant of the primary phase of VO_2_; MBP, microcirculatory blood perfusion; AVBC, average velocity of blood cells; MDA, malondialdehyde; GSH-PX, glutathione peroxidase; VEGF, vascular endothelial growth factor; ACh Tmax, acetylcholine time to maximum perfusion.

### 3.1 Neurohumoral Regulation of Capillary Structure

Regular physical activity promotes increased nitric oxide (NO) secretion, which facilitates vasodilation, enhances microvascular blood flow perfusion, and regulates hemodynamics. These changes constitute the physiological foundations for the improvement of microcirculation function through physical activity. Additionally, supplementing with L-arginine after acute resistance exercise significantly increases muscle blood flow perfusion and enhances skeletal muscle glucose uptake capacity. This effect is likely due to the increased NO secretion driven by L-arginine, resulting in vasodilation and improved skeletal muscle metabolism [10]. Conversely, injecting the non-specific synthase inhibitor N(G)-monomethyl-L-arginine (L-NMMA) into healthy subjects inhibited skeletal muscle metabolism and prevented changes in local blood flow. This indicates that NO plays a role in mediating skeletal muscle glucose uptake and utilization during exercise.

Nitric oxide synthase (NOS), comprising NOS1 (neuronal nitric oxide synthase, nNOS), NOS2 (inducible nitric oxide synthase, iNOS), and NOS3 (endothelial nitric oxide synthase, eNOS), use the amino acid L-arginine as a substrate to synthesize NO [59]. Physical activity can upregulate the expression levels of the entire NOS family. In particular, NOS3 expression is closely associated with exercise-induced phosphorylation of AMP-activated protein kinase alpha (AMPKα). This phosphorylation synergistically regulates peroxisome proliferator-activated receptor gamma coactivator 1-alpha (PGC-1α) and mitochondrial biogenesis, thereby enhancing skeletal muscle metabolic function.

A study on healthy mice subjected to swimming interventions has demonstrated significant activation of eNOS expression in the heart, accompanied by increased mitochondrial density and number, as well as improved skeletal muscle metabolic activity [56]. However, such adaptive changes were not observed in *NOS3* gene knockout mice not expressing the eNOS protein [60]. In mice with impaired *eNOS* gene expression, an upregulation of nNOS gene expression was observed. Different localizations of nNOS contribute uniquely to the effects of enhanced physical activity: Golgi-associated nNOS maintains microvascular cell structural integrity, membrane-associated nNOS in muscle cells regulates oxygen transport and utilization, and cytoplasmic nNOS modulates the balance of glucose breakdown and utilization during physical activity. This regulation enhances muscle mass and delays fatigue. An increase in nNOS expression activation can be observed after just 10 days of physical activity intervention [61].

The bioavailability of NO is determined by the balance between its enzymatic production and degradation by reactive oxygen species (ROS) [62]. Cardiovascular risk factors, such as oxidative stress can induce endothelial dysfunction, thereby reducing NO bioavailability and impairing vasodilation capacity. This reduction in vasodilation can lead to insufficient blood flow to meet myocardial oxygen demands, potentially leading to myocardial ischemia and angina [63]. Enhanced NO bioavailability increases guanylate cyclase (GC) activity, promoting the synthesis of cyclic guanosine monophosphate (cGMP). This signaling cascade increases the phosphorylation of myocardial troponin and reduces intracellular Ca^2+^ levels in vascular smooth muscle, facilitating muscle relaxation. Additionally, NO indirectly modulates oxygen transport and energy metabolism by regulating blood flow and hormonal levels, thereby delaying the onset of skeletal muscle fatigue.

NO works in synergy with various hormones to regulate microcirculation. Notably, NO can mediate hormone production and sensitivity. In healthy subjects supplemented with L-arginine, a significant enhancement in skeletal muscle glucose metabolism was observed, without affecting insulin levels [16]. This metabolic improvement is attributed to increased NO secretion. In mice with impaired NO expression, blood glucose levels were significantly lower during exercise compared to healthy mice, and post-exercise hypoglycemia was also noted. Thus, NO likely regulates glucose metabolism by inducing insulin secretion and sensitivity. Endothelin-1 (ET-1) is an endogenous long-acting vasoconstrictor that, in coordination with NO, plays a critical role in regulating microvascular contraction and cardiovascular homeostasis. In individuals engaged in long-term aerobic exercise, NO levels gradually increase while ET-1 levels decrease [12]. This may be because NO inhibits ET-1 synthesis and release and reduces ET-1 levels and bioactivity by activating endothelin-converting enzyme expression.

Endocrine feedback can regulate both the expression and activity of NO and NOS. Growth hormone treatment of endothelial cells promotes eNOS gene expression, thereby increasing NO secretion and significantly reducing ROS levels in these cells [1]. Numerous studies have demonstrated that physical activity can improve insulin secretion and sensitivity [17, 52]. Enhanced insulin secretion and activity can feedback stimulate eNOS phosphorylation, promoting further NO secretion. Maintaining normal insulin levels protects microvascular endothelial cells, reduces oxidative stress and inflammatory factors, and prevents endothelial dysfunction [17]. While NO regulates ET-1, ET-1 can also influence NO synthesis and release. For example, ET-1 can activate protein kinase C, inhibiting NOS activity and reducing NO secretion [12]. Additionally, ET-1 can stimulate vascular smooth muscle cells to produce ROS, thereby reducing NO bioavailability.

In addition to NO-regulated microvascular dilation, capillary proliferation is another key factor in the improvement of microcirculatory function due to physical activity. This process is governed by the synergistic control of pro-angiogenic and anti-angiogenic signals. Vascular endothelial growth factor (VEGF) is the most potent known positive regulator that increases capillary numbers. VEGF promotes the division of microvascular endothelial cells, increases angiogenesis, and enhances microvascular permeability [20]. During physical activity, the mechanical stress from increased blood flow enhances the expression of vascular endothelial growth factor A (VEGFA) mRNA. The increased secretion of VEGF then acts on VEGF receptors on capillary endothelial cells, stimulating capillary formation [19, 37].

### 3.2 Reducing Levels of Inflammatory Factors

Inflammatory cytokine levels are closely linked to the pathogenesis of autoimmune diseases, with obesity, particularly abdominal fat, being a significant factor. Numerous studies have shown a connection between abdominal fat, perivascular fat, and the mechanisms of chronic inflammatory diseases [15, 18, 26]. Mice fed a high-fat diet show a significant decline in microcirculation and a marked increase in plasma inflammatory markers, alongside decreases in the levels of protective factors like adiponectin and irisin [15]. Six weeks of running exercise were shown to reduce the levels of inflammatory cytokines, including interleukin-13 (IL-13), interleukin-17 (IL-17), and interleukin-1α (IL-1α) in mice on a high-fat diet [15]. Another study investigating exercise interventions in obese mice reported similar effects [18], likely due to reduced neutrophil infiltration, which subsequently decreases macrophage infiltration and chemokine activity in perivascular tissues.

Physical activity can enhance vascular permeability, facilitating leukocyte migration and inflammatory cell infiltration. Additionally, exercise inhibits the production of inflammatory cytokines such as tumor necrosis factor-alpha (TNF-α) and interleukin-1 beta (IL-1β), thereby attenuating the overall inflammatory response [15]. It is noteworthy that high-intensity endurance exercise can exacerbate systemic inflammation and immune suppression, while regular, moderate physical activity has anti-inflammatory effects. There is also a potential interaction between physical activity and inflammatory cytokines like interleukin-6 (IL-6) and interleukin-8 (IL-8) [38]. Interestingly, NG-nitro L-arginine methyl ester (L-NAME) supplementation significantly reduces the activation of mRNA for IL-6 and *CXCL8* (the gene coding for IL-8) during exercise, indicating a potential link between NO and inflammatory cytokines [64].

In conclusion, physical activity can reduce levels of inflammatory cytokines from adipose tissue, endothelial cells, and skeletal muscle. By synergizing the effects of NO and inflammatory cytokines, physical activity mitigates damage to microvascular endothelial cells.

### 3.3 Improving Oxidative Stress in the Organism

The bioavailability of NO is shaped by the balance between enzymatic and non-enzymatic reactions that generate and scavenge NO through ROS. Cellular levels of ROS are influenced by mitochondrial nicotinamide adenine dinucleotide phosphate (NADPH) and xanthine oxidase activity, as well as the body’s antioxidant stress response to clear ROS [26]. Typically, mitochondria produce small amounts of ROS during respiration, which are neutralized by cellular antioxidant enzymes. However, in patients with chronic disease, metabolic abnormalities lead to excessive ROS production within mitochondria alongside impaired ROS clearance [48]. This surplus of ROS can compromise endothelial function and damage the cardiovascular system.

Excess superoxide in the body not only directly inhibits NO bioavailability but also promotes eNOS uncoupling, which further increases the production of superoxide instead of NO [27, 48]. Regular physical activity reduces the activity of ROS-generating enzymes and enhances endogenous antioxidant protection, thus decreasing levels of ROS and superoxide, and enhancing NO bioavailability. Interestingly, following acute high-intensity resistance exercise, vascular dilation function is impaired in sedentary individuals, whereas individuals with a history of regular physical activity maintain acetylcholine (Ach)-mediated endothelium-dependent dilation [35]. At this point, the predominant vasodilatory mediator may shift from NO to H_2_O_2_, suggesting that H_2_O_2_ can compensate for reduced NO bioactivity in vasodilation.

The signaling pathway involving advanced glycation end-products (AGE) and their receptor (RAGE) is associated with oxidative stress. Specifically, AGE levels closely linked to RAGE expression, hepatic stellate cell (HSC) activation, and microcirculatory dysfunction. Activation of HSCs may promote fibrogenesis, stimulated by connective tissue growth factor and exacerbated by elevated levels of inflammatory cytokines along with oxidative stress, accelerating liver fibrosis [33]. Physical activity mitigates these effects by reducing AGE-RAGE expression levels, decreasing HSC activation, enhancing vitamin A deposition, and lowering alpha-smooth muscle actin expression, all of which contribute to improved microcirculatory function [33]. Interestingly, physical activity reduces white adipose tissue (WAT) leukocyte chemotaxis and increases microvascular blood flow in type 2 diabetic mice, illustrating the mechanisms by which physical activity can ameliorate microcirculatory dysfunction by modulating oxidative stress [15].

High blood glucose, abnormal lipid profiles and elevated inflammation levels can increase ROS generation, and consequently elevate oxidative stress [28]. Physical activity significantly enhances gene expression at the level of mRNA for antioxidant enzymes including superoxide dismutase-1 (SOD-1), superoxide dismutase-2 (SOD-2), and glutathione peroxidase, while showing no significant effect on heme oxygenase (decycling) 1 (Hmox1) [10]. By activating compensatory defensive responses, physical activity helps to regulate oxidative stress levels and facilitates the repair of microvascular damage [26].

### 3.4 Improving Vascular Mechanical Stress Dynamics

Endothelial cells play a crucial role in controlling vascular tone. Exercise-induced increases in blood flow increase mechanical stress on endothelial cells, which promotes NO secretion to regulate hemodynamics, thus playing a fundamental role in vascular health. However, the specific physiological mechanisms underlying this process remain unclear. A study conducted *in vitro* has demonstrated that the application of shear stress to endothelial cells leads to eNOS activation, potentially through the phosphorylation of platelet endothelial cell adhesion molecule-1 (PECAM-1). However, several human case studies have reported enhanced eNOS phosphorylation with increased blood flow induced by exercise, without altering PECAM-1 phosphorylation [14, 36]. The study primarily investigated changes in PECAM-1 phosphorylation following sustained exercise (20 minutes of passive limb movement, 50 minutes of cycling, 30 minutes of grip exercise), potentially overlooking its dynamics during exercise [36]. Furthermore, endothelial cells cultured *in vitro* are subjected to fluid shear stress (1.2 Pa), while the shear stress induced by exercise in human studies is markedly lower (approximately 0.4 Pa) [65]. This discrepancy suggests that differences in PECAM-1 phosphorylation may relate to variations in the intensity, pattern, and duration of shear stress experienced during physical activity [65].

Future research should investigate the phosphorylation changes of PECAM-1 during various exercise stages and types of exercise. This investigation should determine whether increased microvascular shear stress and microcirculatory changes associated with exercise are related to PECAM-1 activity. Another hypothesis is that exercise decreases the expression of NADPH oxidase II and SOD-2, with vascular shear stress playing a crucial role in mediating adaptation to exercise. Furthermore, exercise promotes the upregulation of key genes such as *NOS3* and Ca^2+^-dependent *KCNN4* (potassium calcium-activated channel subfamily N member 4) channels, significantly enhances cGMP protein activity, and augments Ach-induced vascular dilation effects. It also leads to a notable increase in microvascular macromolecule permeability.

### 3.5 Microcirculation under Hypoxic Conditions

Hypoxic interventions improve microcirculation more effectively than normoxic physical activity, possibly due to unique hypoxia-induced effects [66]. Hypoxic environments activate hypoxia-inducible factors (HIF), promoting mRNA expression of *VEGFA* and lead to increased capillary angiogenesis. Six weeks of hypoxic training increases mRNA and protein levels of silent information regulator 2 homolog 3 (Sirt3), thereby preserving endothelial cell dilation function [13]. Sirt3, a nicotinamide adenine dinucleotide (NAD)+-dependent protein deacetylase, plays a crucial role in regulating various mitochondrial functions and signaling factors. It enhances the expression of CD31 and VEGF at the protein level, thereby improving the quantity and functionality of microcirculatory capillaries [67]. Additionally, Sirt3 increases the tolerance of skeletal muscle mitochondria to hypoxia, enhances mitochondrial metabolism, and promotes capillary growth in skeletal muscle [13]. However, the precise role of Sirt3 in modulating the upstream and downstream signals involved in capillary function is not fully understood. Additional research is needed to elucidate its specific mechanisms and to explore strategies for regulating Sirt3 activity to optimize the effects of hypoxic training.

Interestingly, hypoxia-induced microvascular reactivity changes resemble those observed in conditions of physiological impairment and chronic diseases such as sepsis, type 2 diabetes, and hypertension [13]. Exploring potential connections between these conditions warrants further investigation.

### 3.6 Impact of Physical Activity on Red Blood Cell Deformability in Microcirculation

Decreased red blood cell (RBC) deformability impairs blood flow microcirculation, adversely impacting oxygen transport and utilization in the body, leading to microcirculatory dysfunction and diminished exercise performance. Factors influencing RBC deformability include blood viscosity, oxidative stress, and lactate levels [68]. Notably, research on changes in RBC deformability following acute physical activity yield contradictory results, likely due to differences in assessment criteria along with the intensity, duration and type of physical activity [30]. For instance, isolated elongation index alone may not fully reflect RBC deformability, with maximal deformability being a key parameter for assessing RBC hemorheology. The extent of this influence correlates with exercise intensity: moderate aerobic exercise and functional training improves RBC membrane deformability, while high-intensity training may lead to adverse effects [69]. The specific type of physical activity may also be a contributing factor; for example, cyclists exhibit significantly higher RBC deformability compared to runners, who may exhibit higher tendencies toward RBC fragmentation [69]. However, a study has indicated that RBC deformability does not significantly change before or after a marathon race. There are no substantial alterations in RBC volume or blood viscosity, although there is a tendency towards decreased RBC filterability due to increased osmotic pressure [70].

**Fig. 3.**
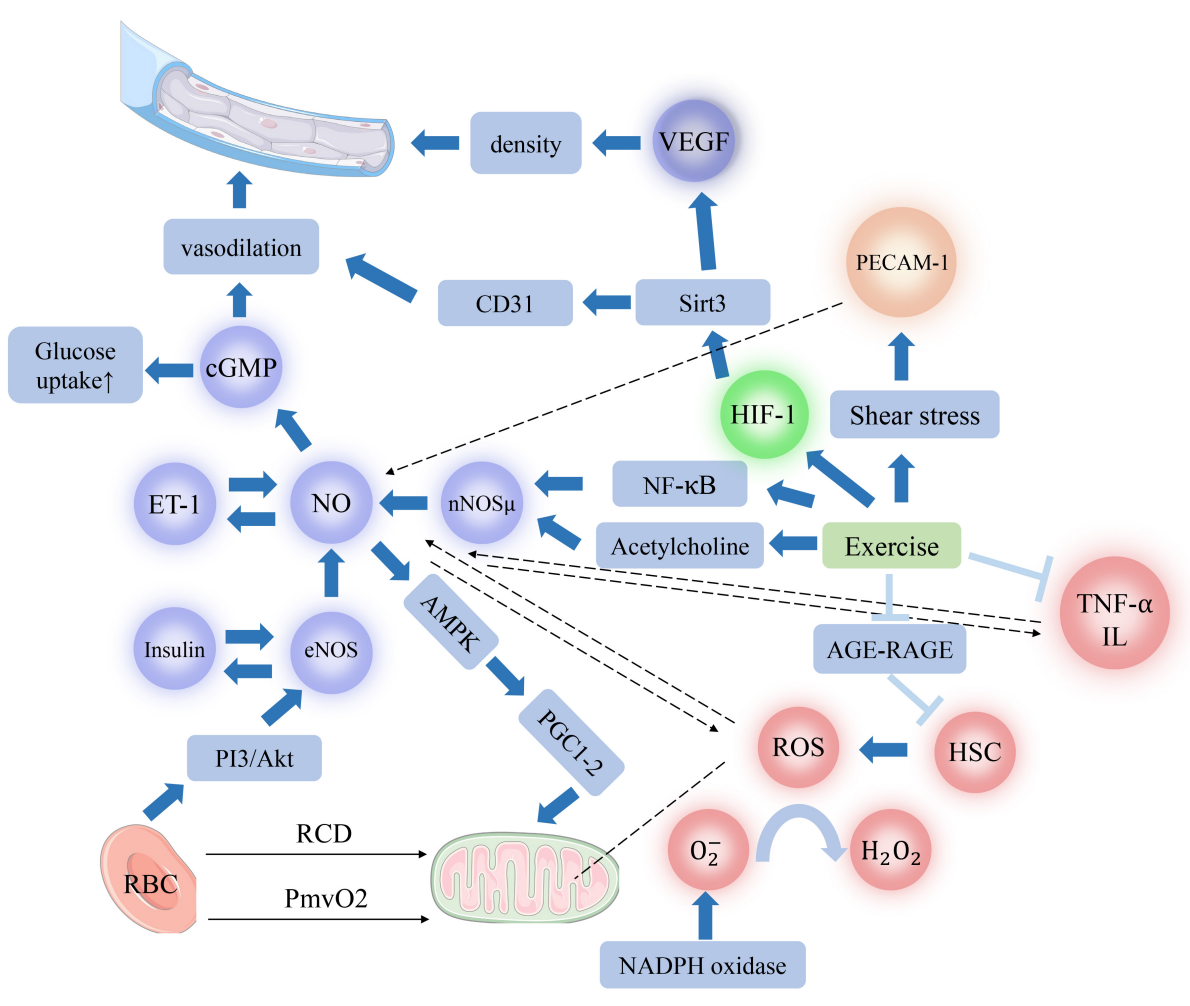
**Physiological mechanisms influencing microcirculation through various types of physical activity**. This diagram illustrates the diverse physiological mechanisms by which different types of physical activity promote or inhibit microcirculation. Note: 🠕 Stimulation; ⤒ Inhibition; ⭫ A correlation exists, yet the precise mechanisms are not fully understood. cGMP, cyclic guanosine monophosphate; ET-1, endothelin-1; NO, nitric oxide; nNOSμ, neuronal nitric oxide synthase (muscle-specific μ isoform); eNOS, endothelial nitric oxide synthase; PI3/Akt, phosphoinositide 3-kinase/protein kinase B; RBC, red blood cell; PO_2_mv, muscle microvascular oxygenation; RCD, regulated cell death; AMPK, adenosine 5^′^-monophosphate-activated protein kinase; PGC1-α, peroxisiome proliferator-activated receptor gamma coactivator-1 alpha; VEGF, vascular endothelial growth facotr; CD31/PECAM-1, platelet endothelial cell adhesion molecule-1; Sirt3, silent information regulator 2 homolog 3; HIF-1, hypoxia inducible factor-1; NF-κB, nuclear factor kappa B; AGE-RAGE, advanced glycation end-products/receptor for advanced glycation end-products; TNF-α, tumor necrosis factor-α; IL, interleukin; ROS, reactive oxygen species; HSC, hematopoietic stem cell; H_2_O_2_, hydrogen peroxide; O^2-^, superoxide anion; NADPH, nicotinamide adenine dinucleotide phosphate.

The physiological mechanisms linking physical activity to RBC deformability remain unclear and warrant further investigation, particularly through comprehensive hemorheological parameter assessments across different forms and intensities of physical activity. Fig. 3 provides a summary of signaling mechanisms regulating microcirculation.

## 4. Conclusions

Improvements to microcirculatory function are closely linked to physical activity, mediated by the regulation of endothelial cell factors such as NO, ET-1, and VEGF. These factors collectively contribute to microvascular dilation and increased capillary density. Interactions between various hormones and endothelial cell factors create feedback loops that synergistically enhance microcirculatory function. Simultaneously, reductions in inflammatory cytokines and oxidative stress levels are linked to increased secretion and bioavailability of NO, resulting in beneficial effects on microcirculation. Research supports that physical activity promotes improvements in hemodynamics and RBC membrane deformability, regulates energy metabolism, and enhances microcirculation. Although limited, available data suggest that hypoxic stimuli influence eNOS activity and NO secretion through multiple signaling pathways, yet their effectiveness remains somewhat limited. Existing research underscores the pivotal role of physical activity in managing various diseases. For instance, exercise interventions in diabetic patients significantly increase insulin secretion and sensitivity. In individuals with chronic heart failure, physical activity enhances skeletal muscle energy metabolism, thereby improving myocardial and microcirculatory function. In hypertensive patients, the increased blood flow from physical exercise-induced shear stress adaptations improves hemodynamics. Physical activity, through a variety of physiological mechanisms, enhances microcirculatory function, positioning it as a promising non-pharmacological approach for combating aging, managing various chronic diseases, and enhancing athletic performance. Overall, exercise enhances the levels of numerous biologically active substances, including endothelial cell factors, hormones, inflammatory cytokines, and ROS, collectively improving the body’s microcirculation.

## Availability of Data and Materials

All data points generated or analyzed during this study are included in this article and there are no further underlying data necessary to reproduce the results.

## Supplementary Material

Supplementary material associated with this article can be found, in the online version, at https://doi.org/10.31083/RCM25302.
